# STATURE ESTIMATE OF CHILDREN WITH CEREBRAL PALSY THROUGH SEGMENTAL MEASURES: A SYSTEMATIC REVIEW

**DOI:** 10.1590/1984-0462/2020/38/2018185

**Published:** 2020-01-13

**Authors:** Joel Alves Lamounier, Nathália Macedo Marteletto, Cristina Amaral Calixto, Marcia Reimol de Andrade, Jacqueline Domingues Tibúrcio

**Affiliations:** a Universidade Federal de São João del-Rei, São João del-Rei, MG, Brazil.

**Keywords:** Anthropometry, Cerebral palsy, Child, Body height, Antropometria, Paralisia cerebral, Criança; Estatura

## Abstract

**Objective:**

To review studies that evaluate the correspondence between the estimate height via segmental measures and the actual height of children with cerebral palsy.

**Data sources:**

Systematic literature review between 1995–2018, guided by the PRISMA criteria (Preferred Reporting Items for Systematic Reviews and Meta-Analyses), in PubMed, BVS, MEDLINE and Lilacs databases. The descriptors, connected by the AND Boolean Operators, were: anthropometry, cerebral palsy, child and body height. The research comprised papers in Portuguese, English and Spanish, with Qualis-CAPES equal or superior to B3 that addressed the question: “Is there any correlation between estimate height by equations and direct height measures in children with cerebral palsy?” 152 studies were recovered and seven were selected. Their methodological quality was assessed by the scale of the Agency for Healthcare Research and Quality (AHRQ).

**Data synthesis:**

Most studies showed no correspondence between estimated and real height. Studies that showed coincidence of the measures contain limitations that could jeopardize the results (sample losses, small samples and exclusion of patients with severe contractures, scoliosis and severe cerebral palsy). Japanese researchers developed an equation which harmoniously aligns the statures; the study comprised only Japanese patients, though.

**Conclusions:**

Given the importance of accuracy in height measures to evaluate infant health, it is crucial to carry out more researches in order to safely establish an association between both estimate and real statures. The development of anthropometric protocols, emerged from such researches, would benefit the follow-up of children with severe psychomotor disabilities.

## RESUMO

**Objetivo:**

Revisar estudos que avaliam correspondência entre a altura estimada por medidas segmentares e a estatura real de crianças com paralisia cerebral.

**Fonte de dados:**

Revisão sistemática da literatura entre 1995 e 2018, guiada pela diretriz Preferred Reporting Items for Systematic Reviews and Meta-Analyses (PRISMA), nas bases de dados PubMed, Biblioteca Virtual em Saúde (BVS), Medical Literature Analysis and Retrieval System Online (MEDLINE) e Literatura Latino-Americana e do Caribe em Ciências da Saúde (LILACS). Os descritores, combinados pelo operador booleano “and”, foram: “anthropometry”, “cerebral palsy”, “child” e “body height”. A pesquisa englobou artigos em português, inglês e espanhol, classificadas pelo Quali-CAPES igual ou superior a B3 e que respondiam à questão guia: “Existe correlação entre a altura estimada por equações em crianças com paralisia cerebral e as medidas diretas de altura?”. Dos 152 artigos inicialmente recuperados, sete foram selecionados e sua qualidade metodológica foi avaliada pela escala da Agency for Healthcare Research and Quality (AHRQ).

**Síntese dos dados:**

A maioria dos trabalhos não encontrou correspondência entre altura real e estimada. Estudos que exibiram coincidência das medidas apresentaram limitações que poderiam comprometer os resultados (perda de amostra, amostra pequena e exclusão de indivíduos com contraturas severas, escoliose e paralisia cerebral grave). Pesquisadores japoneses desenvolveram equação que apresenta boa concordância entre as estaturas. Contudo, o estudo compreendeu apenas indivíduos japoneses.

**Conclusões:**

Dada a importância da precisão das medidas de estatura para avaliar a saúde infantil, tornam-se necessárias mais pesquisas visando estabelecer, de maneira mais segura, a associação entre a estatura estimada e a real. O desenvolvimento de protocolos antropométricos, resultantes dessas pesquisas, beneficiaria o acompanhamento de crianças com sequelas psicomotoras graves.

## INTRODUCTION

Nutrition, health, socioeconomic status, and psychosocial aspects all interfere with growth, which is a good overall indicator of well-being from the fetus to adolescent stages of life.^1^ Anthropometric assessments allow for the monitoring of health through growth monitoring.

The Brazilian Ministry of Health has incorporated the 2006 and 2007 World Health Organization’s (WHO) growth curves into the Children’s Handbook, providing health professionals with a valuable tool for monitoring the growth of children and adolescents.^2,3^ However, the evolution charts for weight, length, height, and body mass index (BMI) according to gender and age were created based on healthy subjects.^4-6^ As such, they do not include children with severe psychomotor sequelae, in which measuring length or height is difficult due to body contractures and spinal deformities.

In children with motor limitations, as in the case of cerebral palsy (CP), height measurement is currently performed by segmental measurements, as proposed by Stevenson in 1995.^7^ According to the author, height can be estimated by combining the equations he proposes with each measured segment: upper arm length (UA), knee heel length (KH) and tibial length (T). However, it is necessary to know if the measurements estimated by methods such as this reflect actual height.

Thus, this article performs a systematic review of the literature, with the goal of verifying if there is evidence, in the published works on the subject, of the correspondence between height measured by segmental measurements and the real height of children with CP.

## METHOD

For the development of this systematic review study, scientific literature searches were performed from the US National Library of Medicine databases – the National Institutes of Health (PubMed), the Virtual Health Library (*Biblioteca Virtual em Saúde* – BVS), the Medical Literature Analysis and Retrieval System Online (MEDLINE) and Latin American and Caribbean Health Sciences Literature (LILACS). The descriptors and expressions used in the searches were: “anthropometry”, “cerebral palsy”, “child” and “body height”. These are part of the list of Health Sciences Descriptors (*Descritores em Ciências da Saúde* - DeCs) and Medical Subject Headings (MeSH) and were combined through the Boolean operator AND.

This systematic review included original articles in Portuguese, English and Spanish, indexed in the aforementioned databases, with a temporal delimitation between January 1995 and April 2018, which respected the combination of descriptors: “anthropometry” AND “cerebral palsy” AND “child” AND “body height,” and which fit into an observational study. We excluded articles that were repeated in the databases consulted, studies published in journals evaluated with a Qualis-CAPES score of less than B3, and studies that did not answer the guiding question of this review (is there a correlation between estimated height from equations and direct height measurements in children with CP?).

The search for bibliographic references was performed according to the following steps:

- First step: 159 papers were identified in the databases searched.
- Second step: Two articles in the German language were excluded (only the abstracts were in English and, moreover, they did not match the objective of this study). After using the language filter (Portuguese, English and Spanish), 157 references remained eligible.
- Third stage: The period of interest was selected: between January 1995 and April 2018. The lower limit of this interval was chosen according to the year of publication of the equation proposed by Stevenson, which is often used in clinical practice to estimate height in children with CP.^7^ Applying this filter resulted in the exclusion of 41 articles.
- Fourth stage: of the 116 remaining references, 29 were excluded because they were repeated in multiple databases, which left 87 references. From this point on, the articles were selected based on their content compatibility, considering the guiding question, as described in the following steps.
- Fifth step: PICO eligibility criteria (participant, intervention, comparison, and results - outcomes) were followed, as proposed by the Preferred Reporting Items for Systematic Reviews and Meta -Analyses - PRISMA, 2015 in order to create a question that would guide the search according to the objectives of the research.^8,9^ The use of the PICO criteria allowed for the creation of a research question that made the database search more effective, since it focused on the objective of the study and avoided unnecessary analyses. Therefore, when considering the guiding question “Is there a correlation between estimated height from equations and direct measurements of height in children with CP?”, the PICO criteria were: participants - children with cerebral palsy; intervention - estimation of height from equations; control - nonintervention; and results (outcomes) - correspondence between direct height measurements and height estimated by equations. The analysis, directed by the guiding question, was performed by reading the abstracts of the 87 works that remained after the exclusion of 29 repeated references in the databases.
- Sixth step: this step explained the reason for excluding each article analyzed in light of the guiding question. We found articles in several thematic axes that differed from the purpose of this study: 21 articles specifically dealt with BMI analysis, body composition and growth charts in individuals with motor disorders. Another 21 articles addressed nutrition, eating dysphagia, consequences of gastrostomy, eating difficulties and/or the nutritional status of patients with motor impairment. Eight articles addressed CP, but with regard to bone involvement, bone density and the presence of scoliosis in patients. Another eight articles dealt with neural impairment in premature infants, or those with low or very low birth weights. Seven articles referred to motor changes, motor function classification and functional performance in patients with neural impairment. Six articles spoke to physical activity, physical inactivity and energy expenditure in patients with CP. One paper addressed the theme of CP in book chapter format, not fitting, therefore, the selection of articles proposed by the guiding question. The remaining nine studies that were excluded dealt with very specific themes, such as hypopituitarism, vitamin D deficiency, the effects of hippotherapy on gait in CP children, growth analysis of children after dorsal rhizotomy, antibody analysis in CP children, growth hormone treatment, prostaglandin levels and short height in cases of hydrocephaly. In addition, in order to be included in this review, selected articles had to be published in journals with a Qualis-CAPES score equal to or greater than B3.
- Seventh step: Finally, after excluding the 80 references that did not answer the guiding question, seven articles that met the established criteria were selected. These, in turn, were read in full and the most relevant information from each of them was extracted. With this information, tables were made in order to better see and interpret the results.
- Eighth stage: two tables were produced (Tables 1 and 2), which allowed for a synthesis of the most relevant information from each article. To this end, columns were created with the following titles: author and location of the study, sample size, age group, measured segment, measuring instruments, calculation of estimated height, conclusions on anthropometric measurements and limitations of the study.

**Table 1:** Characteristics of anthropometric studies involving segmental measurements.

Author	Location	Sample Size	Age range (years)	Measured segment	Measuring Instruments
García Iñiguez et al.^13^	Mexico	108 with CP	2 to 16	T, KH, and UA	- measuring tape for T - segmometer for KH and UA
Haapala et al.^16^	United States	137 with CP	2 to 25	Height, segmental length measured in decubitus, KH, T and U	- height stadiometer - flexible steel tape measure for T, KH and segmental length - segmometer for KH
Amezquita et al.^17^	Chile	60 with CP	3 to 15	T and KH	- segmometer for KH - measuring tape for T
Kihara et al.^15^	Japan	50 with CP and 38 healthy people	3 to 12	T and body length divided into segments	- tape measure
Teixeira and Gomes^19^	Brazil	14 with CP	0 to 3	Length and KH	- horizontal anthropometer for length - inextensible tape measure for KH
Bell and Davies^14^	Australia	17 with CP and 20 healthy people	5 to 12	KH	- vertical height stadiometer - segmometer for KH
Hogan^18^	Canada	34 with CP	6 to 30	Recumbent length and KH	- segmometer for KH - horizontal stadiometer for recumbent length

CP: cerebral palsy; T: tibial length; KH: length or height from heel to knee; UA: upper arm length; U: ulnar length.

**Table 2:** Analysis of the results of the selected studies.

Author	Calculation of estimated height	Conclusions about anthropometric measurements	Limitations
García Iñiguez et al.^13^	Stevenson Equation*	Estimated height by T and KH were similar. Both differed from that estimated by UA.	BMI (WHO gold standard chart) analysis from estimated height is flawed.
Haapala et al.^16^	Stevenson Equation* if ≤12 years old. Equations of Chumlea et al.** if ≤6 years old. Equation of Gauld et al.*** if ≤7 years old.	There is a flawed agreement between the actual height and the height estimated from the evaluated equations. Height estimation using segmental length seems to be the most reliable method in cases of severe CP, scoliosis or contractures.	Lower limb growth is hypoplastic relative to the upper limbs, resulting in bias in an attempt to predict height. This difference increases with the severity of the CP.
Amezquita et al.^17^	Stevenson Equation*	Estimated length is the same as actual height in sample of Chilean children with CP.	In 40% of the sample it was not possible to perform direct measurements of height.
Kihara et al.^15^	Proposed equations to estimate height based on T. Typical development: Height = CTx3.25 +34.45 [cm] Children with CP: Height = CT × 3.42 +31.82 [cm]	The calculation made from these equations is independent of the presence of scoliosis or joint contracture. KH is not appropriate for estimating height in cases of severe ankle joint contracture.	Study conducted with Japanese people only.
Teixeira and Gomes^19^	Stevenson Equation**	Actual and estimated length correspondence from KH.	Small sample (n = 14). Age range from 0 to 3 years old.
Bell and Davies^14^	Equations of Chumlea et al.** and Stevenson*	Equations have errors in individual analysis: Stevenson Equation* range between -12.7 cm (10%) and +11.8 cm (9%) for children with CP Chumlea Equation: range between -11.3 cm (9%) and +13.3 cm (11%) for healthy children	Small sample (17 with CP and 20 healthy children). The following were disregarded: Children with severe contractures/scoliosis/severe CP.
Hogan^18^	Equations of Chumlea et al.**	Regardless of age, gender or type of CP, KH was considered a good predictor of recumbent length.	Small sample (n = 34). Measurement of recumbent length in children with CP is subject to errors (contractures, spastic movements, tactile defense).

**Figure 1:**
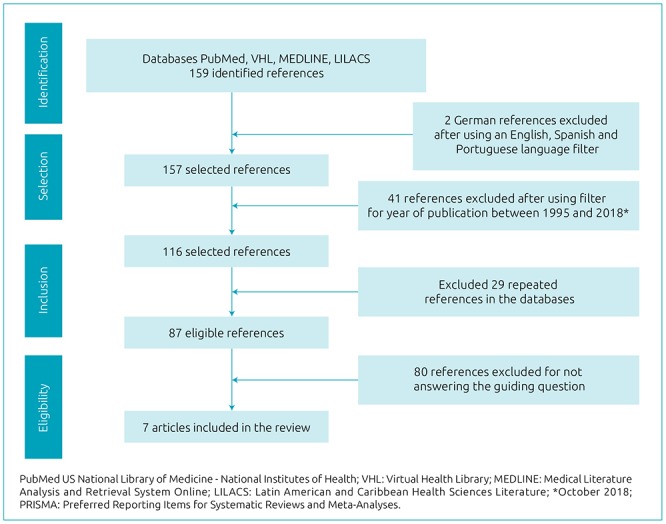
Research flowchart according to Preferred Reporting Items for Systematic Reviews and Meta-Analyses (2009): identification, screening, eligibility and inclusion of scientific articles in the systematic review.
- Tenth stage: after the selection of the articles, a secondary search was made in the bibliographic references of each of the selected articles. However, the articles that answered the guiding question were the same as those found in the primary search. Thus, the discussion of the results was limited to the seven articles located in the initial screening.

*equations to predict height from KH, UA and T of 172 children with CP;^7^ ** equations based on healthy individuals developed for use in persons with reduced mobility (sample of 13,800 healthy children);^11^ *** equation based on estimated height from U in 2,343 healthy individuals with a specified gender; 12 T: tibial length; KH: length or height from heel to knee; UA: upper arm length; BMI: body mass index; WHO: World Health Organization; U: ulnar length; CP: cerebral palsy.
- Ninth step: A flowchart was prepared according to the PRISMA recommendation model, consisting of four steps (identification, selection, eligibility and inclusion), to synthesize the technique of the systematic review performed (Figure 1).

Determining the quality of the methods used in each article during this systematic review was independently performed by two reviewers using the modified quality criteria evaluation scale for observational studies by the Agency for Health Care Research and Quality (AHRQ).^10^ This instrument evaluates studies according to nine criteria (study question, study population, comparability of individuals, exposure or intervention, outcome measurements, statistical analysis, results, discussion and funding), generating a final score from zero to one hundred. Studies with a score below 50 were considered to be low quality. Those of moderate quality received a score between 50 and 66 and those with a score above 66 were classified as having high quality methods. Any disagreements among the evaluators regarding the scores given to the articles were resolved by consensus.

The scope of the work was guided by the checklist of items to be included in the systematic review report proposed in PRISMA^9^

## RESULTS

The steps followed for the selection of the seven articles analyzed in this study are described as a flowchart in Figure 1. The results obtained in this systematic review were summarized in Tables 1 and 2. The results of the methodological quality analysis of the seven articles included in this systematic review are shown in Table 3. According to the evaluation performed, three articles (43%) obtained scores between 50 and 66 and were classified as being of moderate quality. Four articles (57%) had a score higher than 66, and were therefore considered to be of high methodological quality.

**Table 3:** An evaluation of the methodological quality of the articles from the systematic review, based on the areas and features of the Agency for Healthcare Research and Quality for observational studies.

Evaluated criterion	Highest score	García Iñiguez et al.^13^	Haapala et al.^16^	Amezquita et al.^17^	Kihara et al.^15^	Teixeira and Gomes^19^	Bell and Davies^14^	Hogan^18^
Study Question	2	2	2	2	2	2	2	2
Study population	8	8	5	5	5	8	5	8
Comparability between individuals	22	14	14	11	22	11	16	9
Exposure or intervention	11	11	11	11	11	8	11	11
Measurement results	20	15	15	15	15	15	15	15
Statistical analysis	19	8	15	10	15	5	8	10
Results	8	8	8	8	8	3	3	8
Discussion	5	3	5	3	5	5	3	3
Funding and sponsorship	5	0	0	0	0	0	5	0
Total	100	69	75	65	83	57	68	66

## DISCUSSION

Limiting factors - such as the presence of scoliosis, muscle atrophy, the inability to stay upright, spasticity and joint muscle contracture - present in CP patients precluded the application of the technique and the use of normal instruments to measure height. These difficulties are mentioned in most of the articles evaluated in this systematic review. ^11-19^ The most widely utilized technique to overcome these problems was to use the equations proposed by researchers to estimate height based on the measurement of body segments. However, as the analyzed studies suggest, this is a reference standard that is subject to many variations. ^14-16^

Bell and Davies, for example, in 2006 compared children with CP and healthy children using the equations described by Chumlea et al. in 1994 and Stevenson in 1995. There were variations of the order of 9 to 10% between the estimated height and the height obtained through direct measurement. In this study, the Stevenson equation was suitable only for children with mild CP, in whom pronounced body contractures do not occur. ^14^

Kihara et al. conducted a comparative study in Japan between CP patients (with severe contractures and/or scoliosis) and healthy individuals, estimating height from T. The actual height measured could be considered quite accurate, since the researchers made the measurements by dividing the body length into contiguous linear segments that, when added, represented the actual height. Thus, these authors developed regression equations for the calculation of estimated height (from T), which were suitable for both healthy children and those with neural impairment.^15^ This Japanese study also pointed to the fact that children with CP are shorter than children with typical development and they observed significant differences between the height estimated by the Stevenson technique for each measured segment (T, KH and UA). One hypothesis to justify the variability of these measures is highlighted by Haapala et al. According to these authors, there are differences in the growth of body segments, according to the severity of paralysis, which may cause errors in estimating height through segmental measurements.^16^

Amezquita and Bunster, on the other hand, agreed on the direct measurement of height and the height values estimated by the KH and T segments, using the Stevenson equations.^17^ However, in 40% of the sample, it was difficult to perform direct measurements of height in an orthostatic position. This fact represents an important limitation of this study, since these 40% coincide precisely with individuals with moderate to severe paralysis. Thus, an analysis of the equation correspondence only included patients with mild paralysis.

Haapala et al. also investigated the validity of the Stevenson equations, among others, proposed by Chumlea in 1994 and Gauld in 2004. This work demonstrated the validity of equations for estimating height. However, for individuals with a high degree of musculoskeletal and orthopedic impairment, there was poor agreement between actual measurements and height calculations, leading the authors to recommend caution in applying these equations to these individuals.^16^ Corroborating these findings, García Iñiguez et al. observed that estimated height from equations was significantly higher in individuals with spastic paralysis compared to individuals with other types of CP. ^13^

It is also important to highlight the lack of age group stratification in some studies. The separation of measurements for each age is important since, for each phase of child development, there are differences in the limb size proportions. Therefore, the incorporation of adults and children in the same sample, as in the study by Hogan and Haapala et al., may generate bias.

With regard to methodological quality, the articles included in this systematic review were examined according to the criteria of the AHRQ scale, modified by West et al., and obtained scores that rated them as being of moderate (43%) to high (57%) quality.^10^ However, they all had some degree of limitation with respect to the domains evaluated. Certain items contributed to the loss of points in most articles, such as: the lack of information regarding the treatment of confounders,^13,14,16-19^ the lack of a sample calculation or adequate justification for the size of the sample^14-17^ and the lack of consideration of study limitations in the discussion of results.^13,14,17-18^ In addition, the lack of statistical analyses also contributed to reducing the score of the examined articles. Calculating statistical power, for example, was mentioned in only one of the articles.^17^ Most studies did not use modeling or multivariate techniques ^13,14,17,19^ and they did not evaluate confounders. ^13,14,16-19^ With regard to the criteria for presenting the results, only two of the articles evaluated failed to have appropriate effect or precision measurements.^14,19^ None of the studies were blindly quantifying intervention outcomes. Regarding the use of concurrent controls, only two studies presented this investigation model. With regard to the measurement techniques employed, only one of the studies did not use a suitable instrument (KH measurement was performed with a tape measure, which is more prone to errors than a segmometer).^19^ It is also important to point out that, in the investigated articles, due to the specificity of their objectives, no dose-response effects were evaluated. Moreover, because these were all cross-sectional studies, there was no follow-up of the populations’ studied. Only one study received funding or sponsorship.^14^

Finally, as a limitation of this systematic review, we highlight the scarce number of articles on the topic addressed here. In addition, the samples evaluated in these studies were mostly small. Another restriction factor that deserves to be mentioned is the time interval used in the selection of articles (1995 to 2018), which may have contributed to this small number of selected studies, since some of them mention similar works published before 1995. Furthermore, the inclusion of other languages and searches in other databases could increase the number of selected studies, providing new information.

It can be concluded, therefore, that anthropometry provides important information for the monitoring of healthy children with CP, contributing to the tracking of their development. Knowing that there may be differences between actual height and height calculated from equations, it is possible, therefore, that significant discrepancies in the calculation of BMI may occur, since it is based on the ratio between the mass and the squared height of the individual. Thus, slight variations in the estimation of height can generate exponential errors in the calculation of BMI, leading to misdiagnosis and misleading approaches regarding the nutritional status of patients.

This systematic review proposed the objective of verifying, in the literature, studies that showed correspondence between estimated height from segmental measures and the actual height of children with CP. As suggested by most of the studies analyzed, the equations used in clinical practice to estimate the height of children with CP, such as Stevenson’s (proposed in 1995), are more closely matched to real height when applied to individuals with mild paralysis. For children with severe paralysis (with contractures, muscular spasticity and scoliosis), whose measurement of height in the standing position is difficult to measure, the use of methods such as those proposed by Kihara et al. in 2014, which uses equations based on direct measurements of height (obtained by dividing body length into contiguous linear segments) is indicated.

Thus, given the importance of the accuracy of height measurements to assess child health, further research is needed to establish, more safely, the association between estimated and actual height. The development of anthropometric protocols resulting from these studies would benefit the monitoring of children with severe psychomotor sequelae.
